# Retrospective Study of Indications and Outcomes of Open Abdomen with Negative Pressure Wound Therapy Technique for Abdominal Sepsis in a Tertiary Referral Centre

**DOI:** 10.3390/antibiotics11111498

**Published:** 2022-10-28

**Authors:** Francesco Prete, Giuseppe Massimiliano De Luca, Alessandro Pasculli, Giovanna Di Meo, Elisabetta Poli, Lucia Ilaria Sgaramella, Piercarmine Panzera, Francesco Vittore, Antonella Filoia, Fausto Catena, Mario Testini, Angela Gurrado

**Affiliations:** 1 Academic General Surgery Unit, Department of Biomedical Sciences and Human Oncology, Medical School, University of Bari “Aldo Moro”, 70121 Bari, Italy; 2 Emergency and Trauma Surgery Department, Maggiore Hospital of Parma, 43126 Parma, Italy

**Keywords:** abdominal sepsis, open abdomen, damage control surgery, negative pressure wound therapy, acute care surgery

## Abstract

In patients with advanced sepsis from abdominal disease, the open abdomen (OA) technique as part of a damage control surgery (DCS) approach enables relook surgery to control infection, defer intestinal anastomosis, and prevent intra-abdominal hypertension. Limited evidence is available on key outcomes, such as mortality and rate of definitive fascial closure (DFC), which are needed for surgeons to select patients and adequate therapeutic strategies. Abdominal closure with negative pressure wound therapy (NPWT) has shown rates of DFC around 90%. We conducted a retrospective study to evaluate in-hospital survival and factors associated with mortality in acute, non-trauma patients treated using the OA technique and NPWT for sepsis from abdominal disease. Fifty consecutive patients treated using the OA technique and NPWT between February 2015 and July 2022 were included. Overall mortality was 32%. Among surviving patients, 97.7% of cases reached DFC, and the overall complication rate was 58.8%, with one case of entero-atmospheric fistula. At univariable analysis, age (*p* = 0.009), ASA IV status (<0.001), Mannheim Peritonitis Index > 30 (*p* = 0.001) and APACHE II score (*p* < 0.001) were associated with increased mortality. At multivariable analysis, higher APACHE II was a predictor of in-hospital mortality (OR 2.136, 95% CI 1.08–4.22; *p* = 0.029). Although very resource-intensive, DCS and the OA technique are valuable tools to manage patients with advanced abdominal sepsis, allowing reduced mortality and high DFC rates.

## 1. Introduction

Acute, non-trauma cases requiring an open abdomen (OA) approach in the context of damage control surgery (DCS) typically include elective general surgery procedures with unexpected intervening intraoperative complications—most typically significant blood loss during complex gastrointestinal tract surgery—and urgent cases [1]. While intra-abdominal hypertension is a standard indication to use the OA technique as part of a DCS approach following elective surgery [2], intra-abdominal infection is another common indication for the use of the OA technique in complicated elective and acute care surgery [3]. Abdominal sepsis includes a wide range of pathological conditions such as generalized primary or secondary peritonitis or infected necrosis from severe acute pancreatitis. In patients with advanced sepsis, the OA technique enables surgeons to abbreviate initial surgery in patients with severely compromised physiology, allowing relook surgery to control the source of infection, defer intestinal anastomosis until appropriate resuscitation and hemodynamic stability is achieved, and prevent intra-abdominal hypertension leading to abdominal compartment syndrome (ACS) due to important visceral edema [4,5]. The use of the OA technique in abdominal sepsis is increasing worldwide [6], yet it has also been associated with potentially critical complications that can impact mortality rates in frail patients [7].

Few observational studies focused on the OA technique in septic patients [1,3,8], and a limited body of evidence is available on key outcomes of the OA technique, such as mortality and rate of fascial closure, which are needed for surgeons to adequately choose among different therapeutic strategies and select patients.

When the skin and fascia are not closed after laparotomy, the OA technique requires a temporary abdominal closure (TAC) [9]. Several methods of TAC have been described [10,11,12,13], with prospective and retrospective observational studies, and at least one randomized study, showing rates of delayed primary fascial closure around 90% using negative pressure wound therapy (NPWT) [14,15,16].

We conducted a retrospective study to evaluate in-hospital survival and factors associated with mortality in acute, non-trauma patients treated with the OA technique and TAC with NPWT, in the context of DCS for sepsis from abdominal disease. Secondary outcomes were postoperative complications, primary closure rate, time to abdominal closure, and length of stay in the ICU.

## 2. Results

Fifty patients were treated using DCS with the OA technique for abdominal sepsis at the Division of Surgery “V. Bonomo” between February 2015 and July 2022. Demographic data are reported in Table 1. About half of patients (54%) were male, the mean age was around 6o years, and the mean BMI was 28 kg/m^2^, with a quarter of the patients being obese. Almost 90% of patients had at least one comorbidity, with 40% of them presenting three or more conditions. Comorbidities were mainly represented by malignancy (54%, *p* = ns), hypertension and diabetes (34%, *p* = 0.024 and 32%, *p* = 0.011, respectively), and obesity and chronic pulmonary diseases (26%, *p* = 0.001 and 24%, *p* < 0.001). In total, 80% of cases were ASA 3 or greater, with 16 patients ASA 4 (32%, *p* = 0.011). The mean Mannheim Peritonitis Index was 20.8 ± 8.8, and the mean APACHE II was 15.4 ± 8.1. The mean time to surgery was 8.9 ± 2.7 h.

Of the 50 patients, 22 (44%) presented with bacterial peritonitis secondary to anastomotic leakage, 15 (30%) with perforated bowel, 7 (14%) with peritoneal abscesses, and 4 (8%) with infected necrosis from acute severe pancreatitis (Table 2). The mean OA duration was 5.3 +/− 5.4 days, with a mean number of 2.6 +/− 2.7 looks. In all cases, the NPWT TAC technique was used, with mesh-mediated NPWT-assisted closure in three cases (6%). All patients received parenteral nutrition (Table 3).

The overall mortality was 32%. Seven patients (43.8%) died during OA treatment, while nine (56.3%) were deceased after definitive fascial closure, of which six (37.5%) were deceased during the first 30 days from fascia closure. The causes of death were multiorgan failure due to sepsis (75%) and cardiopulmonary complications (25%). The most frequently isolated pathogens from peritoneal swabs collected at DCS were, in order of frequency: *Enterococcus* spp., enteroadhesive/enterotoxic *E. coli*, and *Klebsiella*.

Among the surviving patients, 97.7% of cases reached definitive fascial closure.

Of these, three patients (7%) required a prosthetic mesh (bioabsorbable, intraperitoneal in all cases). Postoperative complications occurred in 58.8% of cases. After definitive fascial closure, the 30-day reintervention rate was 11.6%. The reasons were represented by hemorrhage (one case), tertiary peritonitis (one case), multiple peritoneal abscesses (one case), bowel ischemia (one case), and urinary fistula (one case). Two patients (4.7%) developed an entero-atmospheric fistula (EAF), one of which died before OA closure with multiple small bowel EAFs, more than 30 days after index surgery. The patient who survived developed an EAF of the duodenum (seventh PO day) during the OA procedure for necrotic pancreatitis, and was a frail patient with dense visceral adhesions (frozen abdomen). The EAF prevented fascial closure and, with treatment, developed into an entero-cutaneous fistula. The mean ICU LOS was 19.1 +/− 20.7 days.

There was no significant difference in the interval time before surgery among patients surviving (7.3 +/− 2.7 h) and those who died (7.9 +/− 1.8h) (*p* = 0.443), nor for patients who experienced a complication (9.48 ± 7.02 h) vs. those who did not (6.71 ± 3.38 h) (*p* = 0.237).

At univariable analysis, factors associated with mortality were age (*p* = 0.009), ASA IV status (<0.001), Mannheim Peritonitis Index >30 (*p* = 0.001), and APACHE II score (*p* < 0.001) (Table 4).

At multivariable logistic regression analysis, an increase of 1 point of the APACHE II score was associated with an increase in the OR of perioperative mortality by 2.136 (95% CI 1.08–4.22; *p* = 0.029).

## 3. Discussion

This study shows that the OA technique in septic patients is feasible and resource-demanding, with multiple reoperations and a prolonged stay in the ICU and the hospital. Two-thirds of the patients treated with the OA technique survived, despite high morbidity. APACHE II was a predictor of mortality. The NPWT is a feasible form of TAC with few serious adverse effects, and it allows a high rate of fascial closure.

The presence of multiple comorbidities was a common finding given the age distribution of patients, shifted to older age groups. Comorbidities were mostly represented by neoplastic, cardiovascular, metabolic, and pulmonary diseases, and were more frequent than in other series [6,17]. The mean BMI was high, and more than one-quarter of patients (26%, *p* = 0.001) presented obesity (BMI > 30 kg/m^2^) compared to a recent study [8].

These characteristics portray a cohort of complex and frail patients, which is reflected in an overall ASA score of III or greater in the majority of participants (88%). High mean values of the ASA score, MPI, and APACHE II confirm the physiological impairment of patients included in this study, with impact on survival [6,18].

The mortality rate observed in our series is consistent with the previously published literature [17]. Previous evidence shows that DCS and the use of the OA technique in elective surgery patients has been associated with a similar mortality rate (35%), likely reflecting the significant blood loss and similar underlying comorbidities (>50% of patients with cancer) [1].

Specific OA treatment variables, such as OA duration, number of looks, and Björck classification, did not show significant differences between survivors and non-survivors, as in previous studies [8]. However, an issue in understanding the effect of treatment on mortality during OA is that clinical indications for OA are composite and the current prognostic scores are dominated by organ dysfunction and are insufficient to adequately select patients who can benefit from the OA technique [19]. In this study, the univariable analysis showed three variables independently associated with mortality in patients with abdominal sepsis (age, ASA 4 status, MPI > 30, and APACHE II score), while in the multivariable analysis, only the APACHE II score resulted in a predictor of in-hospital mortality, in line with the findings of a recent systematic review [20].

Mortality has been shown to be contributed to by the complex and difficult management of these patients in the ICU [21,22].

The mean duration of both the OA and the ICU stay were greater than in a recent, larger study [8], despite the mean MPI and APACHE II scores being similar. The longer OA treatments and ICU stays might be attributable to the higher proportion of obese patients treated in this study, as recent evidence from the International Register of Open Abdomen (IROA) supports [23].

The high overall complication rate (58.8%) found in our series is higher than in 402 prospectively collected patients from IROA (38% during OA and 49.5% after closure), although different etiologies, including trauma and vascular emergencies, making up to one-third of included cases may partly explain this difference [17].

In this study, the entero-atmospheric fistula rate (2.2%) was notably lower than previously published, which is from 5.7 to 17.2% in non-trauma patients [24]. Entero-atmospheric fistulas are a serious complication of the OA technique, with a high related mortality rate known to be up to 30–60% [25,26,27]. Even though the natural history and predictors of EAF formation in OA are largely unknown, we could speculate that our low EAF rate may be related to a relatively short OA duration (5.3 days), as prolonged OA and especially an increased number of re-explorations may increase the risk for EAF and frozen abdomen, as well as increase complications [17].

Failure to achieve fascial closure and the development of an entero-cutaneous fistula has been noted to further compromise the nutritional status of patients, contributing eventually to the mortality rate. In fact, EAF is very difficult to control, and patients face complex wound care and compromised nutritional status.

Based on the data from the literature [28,29] and from recent guidelines recommending that primary abdominal closure be performed within the first 8 days of treatment [30], we remain committed to obtaining fascial closure during the first 7–10 days.

The definitive closure rate shown in our series is higher than the one reported in the literature [31,32,33]. DFC ranged from 3.2 to 100%, with an overall weighted closure rate of 50.2%, in a systematic review showing evidence from 63 series [24]. Of note, there was heterogeneity among the included studies, which included patients who died during OA among those in which fascia was not closed.

The use of negative pressure systems, characterized by a greater efficacy in terms of definitive fascial closure [24,33], may be regarded as a key element positively impacting on the 97.7% DFC rate in this series. Negative pressure wound therapy with continuous fascial traction has been suggested as the preferred technique for temporary abdominal closure [30]. With incremental experience gained in the management of OA treatment, we tend to use every chance of VAC dressing change to isolate the fascial edges and proceed to progressive closure with sutures as early as possible in OA treatment.

There are several limitations to this study. The retrospective design implies a risk of misclassification of patient data obtained from multiple databases. The relatively low number of cases from a statistical point of view increases the chance of a statistical error. Nevertheless, based on the present results, treating patients with the OA technique is feasible and has the potential to reduce mortality from advanced abdominal sepsis. NPWT seems to be a feasible form of TAC with few serious adverse effects. Most patients had their OA closed with primary fascial sutures, while a minority was in need of more advanced reconstructions of the abdominal wall.

DCS and OA techniques are valuable tools for the management of patients with advanced sepsis of abdominal origin, though their use is a very resource-intensive decision as patients may require several operations and may have prolonged ICU stays. These complex cases need constant and coordinated care by a dedicated multidisciplinary team including surgeons, anesthetists, operating room staff, and intensivists, among others. Despite its complexity, this approach may be associated with reduced mortality in these critically ill patients, and surgeons should consider the use of this technique selectively in urgent situations or elective operations with complications. The careful selection and management of OA patients will avoid prolonged treatment and facilitate early DFC. Future research should focus on the development of a prognostic model for patients who are potential candidates for OA treatment. Further, prospective studies comparing different methods of temporary abdominal closure among multiple centers in the context of DCS may help clarify the role of NPWT in achieving definitive fascial closure while complementing OA treatment.

## 4. Materials and Methods

### 4.1. Methods

We designed a single-center, retrospective observational study to answer the following research question: in a population of patients who underwent damage control surgery using the open abdomen technique by negative pressure wound therapy for sepsis from abdominal disease, what are the outcomes of DCS using the OA technique by NPWT for intra-abdominal sepsis?

The study was conducted at the “V. Bonomo” Academic General Surgery Unit, Policlinico di Bari, a tertiary University Hospital in Bari, Italy.

Patients undergoing DCS with OA treatment with a diagnosis of abdominal sepsis (secondary generalized peritonitis due to intestinal perforation, necrotizing infected acute severe pancreatitis, multiple abdominal abscesses) and/or septic shock from February 2015 to July 2022 were reviewed.

Among 134 patients treated with abdominal NPWT, 60 consecutive patients had OA treatment by NPWT after DCS procedures for emergency indications other than trauma. Of these, in fifty cases the indication included intra-abdominal sepsis.

The OA technique was adopted in cases of a massive grade of peritoneal contamination; when patients’ severe comorbidities or physiological derangement did not allow the patient to sustain a prolonged operative duration; a projected duration of procedure over three hours; visceral edema observed at laparotomy anticipating high intra-abdominal pressure; hemodynamic instability or severe acidosis; planned relook; incomplete or planned staged control of source of intra-abdominal sepsis; and septic shock due to abdominal peritonitis (defined according to the 3rd International Consensus Definitions for Sepsis and Septic Shock) [4].

The Institutional Review Board approved the study design (reg. n. 2022/7467). This research complied with Ethical Standards and informed consent was obtained from all patients.

Deidentified data were collected from the patients’ medical records and the surgical procedure registry. Information about organ failure, Acute Physiology and Chronic Health Evaluation II (APACHE II) [34], and details of intensive care admission, including type of nutrition, were collected from the ICU registry.

In patients with OA treatments, commercial kits for Vacuum-Assisted Closure (V.A.C.) therapy (Ab Thera, KCI International, San Antonio, TX, USA or RENASYS AB Kit, Smith & Nephew, Memphis, TN, USA) were used for NPWT, applying a continuous negative pressure between 25 and 125 mmHg.

After establishing the OA treatment at the index operation, the dressing was changed every 48–72 h in the operating room or in the ICU. For patients who needed more than one dressing change, the routine care was NPWT only, or by vacuum-assisted wound closure (VAWC). The open abdomen was initially managed with a commercially prepared sponge device. Aggressive diuresis was initiated after successful resuscitation and normothermia to facilitate closure. On the occasion of each dressing change every 48 h, sequential attempts at fascial closure were made. Some patients were treated with vacuum-assisted wound closure and mesh-mediated fascial traction (VAWCM).

Patients who developed an entero-atmospheric fistula (EAF) were treated either with negative pressure therapy or mediated through a dedicated enteric fistula effluent diversion device chimney, made by KCI (“Wound Crown”, “Fistula Funnel”, Acelity San Antonio, TX, USA), over the intestinal opening, preventing intestinal fluid contamination of the abdominal cavity.

At the end of the OA treatment, the fascia was closed with interrupted vycril sutures. If tension-free closure was impossible due to loss of domain, reconstruction with biosynthetic mesh was performed with Bio-A (Gore Inc., Newark, DE, USA) in an intraperitoneal position.

### 4.2. Measures of Outcome

The primary outcomes were survival, defined as rate of in-hospital death and postoperative complications. The secondary outcomes were rate of fascial closure by NPWT, with or without mesh positioning, and rate of surgical interventions other than vacuum-assisted closure (VAC) dressing positioning during postoperative course of index DCS procedure.

Classification of the OA was conducted according to the amended Björck classification [35] at the second look, as previously described [8].

### 4.3. Statistical Analysis

Categorical variables were presented as numbers and percentages, whereas continuous variables were presented as mean± standard deviation. Association analysis between mortality and variables potentially affecting outcome (gender, age, BMI, comorbidities, ASA score, MPI, APACHE II, time to surgery, amended Björck grade at second look, number of surgical looks, OA duration, and ICU LOS) was carried out. Univariable associations between dichotomous and categorical outcome variables were examined using the Chi-square test/Fischer’s exact test as appropriate. The ANOVA Kruskal–Wallis’ test was used to compare differences in continuous variables between groups. In-hospital mortality and the rate of postoperative complications were assessed in relation to pre- and intra-operative disease and patient characteristics using multivariable logistic regression analysis. A multivariable analysis was carried out with a binary logistic regression model in stepwise backward mode. Statistical analysis was conducted using SPSS^®^ ver. 22.0.0 software (IBM, Armonk, NY, USA), with significance set at <0.05.

## Figures and Tables

**Table 1:** Demographics and treatment variables.

Patients			*n* (%), Mean +/− SD
All			50 (100)
Male gender			27 (54)
Age			59.5 +/− 14
BMI			27.6 +/− 3.4
Comorbidities			
Hypertension			17 (34)
Malignancy			27 (54)
Ischemic heart disease			5 (10)
Diabetes			16 (32)
Pulmonary disorder			12 (24)
Obesity (BMI ≥ 30)			13 (26)
Immunological disorder			1 (2)
Neurological disorders			1 (2)
Liver failure			4 (8)
Renal failure			4 (8)
Psychiatric disorder			4 (8)
Neurological disorder			1 (2)
None			6 (12)
Other			10 (20)
	Multiple comorbidities median (range)		2 (0–6)
	3+ comorbidities		20 (40)
ASA classification	I		1 (2)
	II		9 (18)
	III		24 (48)
	IV		16 (32)
ASA ≥ III			40 (80)
Mannheim peritonitis score			20.8 +/− 8.8
APACHE II score			15.4 +/− 8.1

**Table 2:** Indications for OA.

Primary or Underlying Condition Leading to Surgery		*n* (%)
Malignant disease	Colorectal cancer	14 (28)
	Bladder/prostate cancer	5 (10)
	Gynecological cancer	5 (10)
	Pancreatic cancer	2 (4)
	Pelvic sarcoma	1 (2)
	Lymphoma (ischemic bowel)	1 (2)
Benign disease	Diverticular disease	5 (10)
	Pancreatitis	4 (8)
	Ventral hernia (adhesions)	4 (8)
	Inflammatory bowel disease	3 (6)
	Bowel obstruction	2 (4)
	Intestinal adhesions	2 (4)
	Bowel ischemia	1 (2)
	Cholecystitis	1 (2)
Procedure being conducted prior to or at OA index operation		
	Emergency laparotomy with peritoneal drainage (including debridement, bowel resection or stoma formation)	13 (26)
	Adhesiolysis (including bowel resection or stoma formation)	7 (14)
	Colon/Rectal cancer resection	11 (22)
	Hartmann reversal	5 (10)
	Bladder/prostate cancer resection	5 (10)
	Gynecological cancer resection	4 (8)
	Pancreatic cancer resection	2 (4)
	Primary ventral hernia repair	1 (2)
	Miscellaneous procedures	2 (4)
Condition indicating DCS and OA at index operation		
	Peritonitis secondary to anastomosis leakage (intestinal, ureteral, pancreaticobiliary)	22 (44)
	With associated bleeding	2 (4)
	Infected pancreatic necrosis	3 (6)
	With associated bowel perforation	1 (2)
	Peritonitis secondary to bowel perforation	15 (30)
	Peritoneal abscess	7 (14)
Indication for DCS/OA		
	Abdominal contamination/persistent source of peritonitis/planned second look	38 (76)
	Extensive visceral edema	7 (14)
	Severe physiological derangement	3 (6)
	Deferred anastomosis	2 (4)

**Table 3:** Postoperative outcomes.

Outcome Type	Specific Outcome	Outcome Subclassification	*n*/tot (%), Mean +/− SD
Perioperative outcomes			
	OA duration (days)		5.3 +/− 5.4
	Björck classification (at second look)	1A	8 (16)
		1B	10 (20)
		1C	10 (20)
		2A	4 (8)
		2B	11(22)
		2C	4 (8)
		3A	1 (2)
		3B	2 (4)
		4	-
In-hospital mortality	Number of looks		2.6 +/− 2.7
	ICU length of stay (days)		19.1 +/− 20.7
	Overall		16/50 (32)
	Before OA closure		7/16 (43.8)
	After fascial closure		9/16 (56.3)
	By cause	Multiorgan failure	12/16 (75)
		Cardiopulmonary complications	4/16 (25)
Definitive fascial closure			42/43 (97.7)
Prosthetic mesh			3/43 (7)
Overall postoperative complications			20/34 (58.8)
Reintervention			5/43 (11.6)
Entero-atmospheric fistula			1/43 (2.3)

**Table 4:** Univariable analysis.

Independent Variable		Survivors	Non-Survivors	
		n, (%); Mean +/− SD		*p*
All		34 (68)	16 (32)	
Male gender		16 (47.1)	11 (68.8)	0.151
Age		56 +/− 13.4	66.9 +/− 12.5	**0.009**
BMI		27.14 +/− 2.8	28.5 +/− 4.5	0.230
Comorbidities				
	Hypertension	12 (35.3)	5 (31.3)	0.778
	Malignancy	18 (52.9)	9 (56.3)	0.827
	Severe heart disease	4 (11.8)	1 (6.3)	0.544
	Diabetes	8 (23.5)	8 (50)	0.061
	Pulmonary disorder	6 (17.6)	12 (37.5)	0.125
	Obesity (BMI ≥ 30)	6 (17.6)	7 (43.8)	0.061
	Immunological disorder	1 (2.9)	0 (0)	0.488
	Neurological disorder	0 (0)	1 (6.3)	0.141
	Liver failure	2 (5.9)	2 (12.5)	0.806
	Renal failure	1 (2.9)	3 (18.8)	0.055
	Psychiatric disorder	3 (8.8)	1 (6.3)	0.754
	None	6 (17.6)	0 (0)	0.073
	Other	5 (14.7)	4 (25)	0.544
	≥3 comorbidities	12 (35.3)	8 (50)	0.322
ASA IV		4 (11.8)	12 (75)	**<0.001**
Mannheim Peritonitis Index ≥ 30		1 (2.9)	7 (43.8)	**0.001**
APACHE score		11 +/− 4.1	24.9 +/− 5.9	**<0.001**
Time to surgery (hours)		7.3 +/− 2.7	7.9 +/− 1.8	0.443
1A Björck grade at second look		5 (14.7)	3 (18.8)	0.716
Number of looks		2.2 +/− 1.5	3.6 +/− 4.1	0.098
OA duration (days)		5.6 +/− 6.7	9.3 +/− 11	0.144
ICU length of stay (days)		19.5 +/− 17	18.8 +/− 25.5	0.939

## Data Availability

Data on this study is available upon request from University of Bari, Department of Biomedical Sciences and Human Oncology, Academic Division of General Surgery “V. Bonomo”.
